# Brain protein burden is related to intravoxel incoherent motion: PET-MR imaging study

**DOI:** 10.3389/fnins.2026.1841093

**Published:** 2026-06-16

**Authors:** Dimuthu Hemachandra, Kevin Zheng, Sara A. Lorkiewicz, Joseph Winer, Hillary Vossler, Guido A. Davidzon, Elizabeth C. Mormino, Tilman Schulte, Kathleen L. Poston, Eva M. Müller-Oehring

**Affiliations:** 1 Department of Neurology and Neurological Sciences, Stanford University School of Medicine, Stanford, CA, United States; 2 Department of Radiology, Stanford University School of Medicine, Stanford, CA, United States; 3 Wu Tsai Neuroscience Institute, Stanford University, Stanford, CA, United States; 4 Phil and Penny Knight Initiative for Brain Resilience, Stanford University, Stanford, CA, United States; 5 Biosciences Division, SRI International, Menlo Park, CA, United States; 6 Department of Psychology, Palo Alto University, Palo Alto, CA, United States; 7 Department of Neurosurgery, Stanford University School of Medicine, Stanford, CA, United States

**Keywords:** amyloid, brain waste clearance, glymphatic system, intravoxel incoherent motion (IVIM), neurodegeneration, PET/MRI, tau

## Abstract

**Introduction:**

Dysfunction in brain protein clearance mechanisms is thought to contribute to many neurodegenerative diseases, yet non-invasive assessment of these mechanisms in humans remains challenging. This study is the first to examine whether intravoxel incoherent motion (IVIM) diffusion MRI metrics, measures of water diffusion and fluid dynamics, are associated with pathological protein accumulation and cognition in aging individuals, and hence whether they serve as a proxy for brain waste clearance function.

**Methods:**

We analyzed data from 94 participants (*n =* 45 *β*-amyloid positive) who underwent simultaneous PET/MRI scans to calculate three key IVIM metrics: D (true diffusion coefficient), D* (pseudo-diffusion coefficient reflecting perfusion), and *f* (perfusion fraction) within 98 regions of interest. A machine learning model was trained to identify the most informative IVIM features for predicting *β*-amyloid (Aβ) status. Selected features were then evaluated for correlations with protein burden (Aβ and tau) and cognitive performance.

**Results:**

The model identified a subset of 25 key features that effectively predicted Aβ status, achieving a predictive accuracy of 80.0% on unseen data. Regions with important IVIM features aligned with previously identified Aβ-affected regions and showed significant correlations with Aβ burden (r = 0.53, *p <* 0.0001) and tau burden (r = 0.61, *p <* 0.0001). A significant negative correlation was observed between IVIM features and cognitive decline (r = −0.60, *p <* 0.0001). When stratified by Aβ status, this correlation remained significant only in the Aβ-positive group (r = −0.61, *p <* 0.0001), but not in the Aβ-negative group.

**Conclusion:**

IVIM-derived metrics (D, D*, and *f*), which measure water diffusion and perfusion dynamics in the brain, may be valuable non-invasive biomarkers of protein accumulation and associated cognitive decline in the aging human brain.

## 1 Introduction

**Figure 1:**
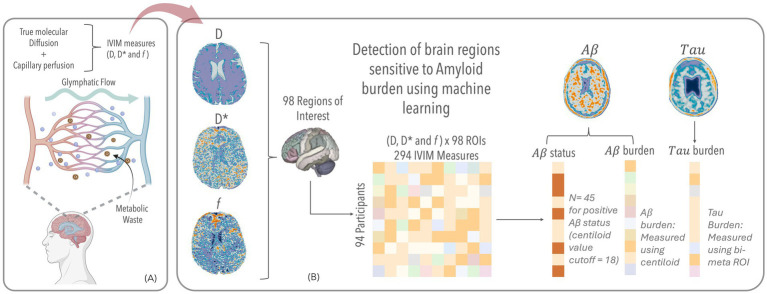
**(A)** Schematic diagram of the glymphatic function and its relation to IVIM measures. Metabolites are released from arterioles and into the extracellular space, while metabolic waste flows toward the peri-venular spaces for glymphatic clearance. Special diffusion-based MRI sequence is used to model intravoxel incoherent motion (IVIM) which offer potential insights into glymphatic flow in the brain. Calculated IVIM metrics. **(B)** Schematic diagram of the workflow of the study. IVIM data from 94 participants were preprocessed to calculate three key metrics: diffusion coefficient (D), perfusion-related diffusion coefficient (D*), and perfusion fraction (f). Mean values for each metric was calculated within 98 ROIs, resulting in a total of 294 features per participants. A random forest-based model was used with leave-one-out cross-validation to identify the most informative IVIM features when predicting the Aβ status. Additionally, using PET data, Aβ burden and Tau burden within those ROIs were also calculated.

Abnormal accumulation of specific proteins, such as *β*-amyloid (Aβ) plaques, neurofibrillary tau tangles, and alpha-synuclein (*α*-syn) aggregates, are the hallmark of age-related neurodegenerative diseases. A mechanism thought to contribute too many neurodegenerative disorders is dysfunction in protein clearance mechanisms (Sundaram et al., 2019; Tarasoff-Conway et al., 2015). There are limited means of non-invasively evaluating the brain waste clearance system *in vivo* in human brains (Kamagata et al., 2024; Kaur et al., 2020; van der Thiel et al., 2023). Recent advances in diffusion magnetic resonance imaging (MRI), specifically the modeling of intravoxel incoherent motion (IVIM), could offer potential insights into glymphatic flow in the brain (Federau et al., 2014) (Figure 1).

IVIM is an advanced MRI technique that allows for the simultaneous assessment of perfusion and diffusion in biological tissues, including the brain. This method is particularly valuable as it can differentiate between the effects of microvascular perfusion and the true diffusion of water molecules, all without the need for exogenous contrast agents (Le Bihan et al., 1988; Wirestam et al., 2001). IVIM employs a series of diffusion-weighted imaging (DWI) volumes with multiple b-values (typically ranging from 0 to 1,000 s/mm^2^), which capture the subtle changes in signal that correspond to both perfusion and diffusion processes (Le Bihan et al., 1988; Federau, 2017).

The IVIM model employs three essential parameters to characterize tissue properties: the true diffusion coefficient (D), the pseudo-diffusion coefficient (D*), and the perfusion fraction (*f*). Measure D quantifies the true diffusion of water molecules within the tissue, serving as an indicator of microstructural integrity and the cellular environment (Edgar and Griffiths, 2009). A higher D value is typically associated with more unrestricted diffusion, which could be indicative of unhealthy tissue (Bodini and Ciccarelli, 2014). In contrast, the D* accounts for the effects of incoherent motion of water molecules within the microvasculature as well as fast diffusion related to interstitial fluid (ISF) and cerebrospinal fluid (CSF) (Wright et al., 2024). This parameter reflects the influence of blood flow and microvascular dynamics on the tissue’s vascular environment. Finally, the *f* estimates the fraction of incoherent motion occurring within a voxel compared to the true diffusion and is critical for understanding the efficiency of fluid flow in the brain (Becker et al., 2018). Together, these parameters provide comprehensive insights into the physiological state and functionality of the tissue.

To acquire accurate IVIM images, it is essential to utilize low b-values (typically ranging from 0 to 100 s/mm^2^) in conjunction with higher b-values (up to 1,000 s/mm^2^ or more). The low b-values are critical for capturing the perfusion effects, while the higher b-values are necessary for assessing the diffusion properties of water molecules in the tissue (Federau et al., 2014).

The glymphatic system is believed to be responsible for clearing metabolic waste from the brain, relying heavily on adequate CSF perfusion (Iliff et al., 2012). Impaired perfusion can lead to reduced clearance efficiency, contributing to the accumulation of neurotoxins, including Aβ and tau proteins (Tarasoff-Conway et al., 2015; Kaur et al., 2020). Recent studies have attempted to connect IVIM measures to glymphatic function by assessing changes in CSF flow dynamics (Wong et al., 2020), though this link has primarily relied on indirect conceptual models of glymphatic clearance, such as enlarged perivascular spaces (Iliff et al., 2012) and white matter hyperintensities (Sabayan and Westendorp, 2021). Critically, research establishing a direct relationship between IVIM metrics and protein accumulation is lacking.

To address this, we conducted a novel investigation using simultaneously acquired positron emission tomography (PET) and MRI scans to evaluate if IVIM measures correlate with Aβ and tau accumulation. Given that glymphatic dysfunction has been proposed as one mechanism contributing to protein accumulation, we hypothesized that IVIM metrics would correlate with protein accumulation as measured by PET. Additionally, we examined whether the IVIM metrics correlates with participants’ cognition to determine the clinical significance of these findings.

## 2 Materials and methods

### 2.1 Participants

We included 94 participants who underwent a diffusion MRI sequence specialized for IVIM modeling from June 2023 through November 2024 as part of their Iqbal Farrukh and Asad Jamal Stanford Alzheimer’s Disease Research Center (ADRC) Imaging Core evaluation. Participants were consisted of neurologically normal older adults and those clinically diagnosed with Alzheimer’s disease (AD) and Lewy body disease (LBD). Clinical motor and cognitive assessments have been described in detail elsewhere (Shahid et al., 2023). Briefly, participants underwent comprehensive clinical evaluation which included neuropsychological testing, neurological examination, and neuroimaging. Clinical diagnoses were determined by a consensus committee as cognitively unimpaired, mild cognitive impairment or dementia. If cognitively impaired, the most likely etiological diagnosis was given according to NACC reporting guidelines. In addition, we used the total Montreal Cognitive Assessment (MoCA) total scores (Nasreddine et al., 2005) as our global cognition measure, where lower values indicate more cognitive impairment.

### 2.2 Scanning parameters

#### 2.2.1 T1 weighted (T1w) imaging

Full brain T1w images were acquired using a 3 Tesla GE SIGNA PET/MRI scanner at the Stanford Lucas Center for Imaging. Scan parameters for the 3D gradient-recalled inversion recovery pulse sequence had repetition time/echo time (TR/TE) = 7.7/3.1 ms, 400 ms inversion time, 11 degrees flip angle, 1 mm slice thickness, and 1 mm spacing, which was acquired simultaneously to the PET data at the beginning of the time window.

#### 2.2.2 Diffusion weighted imaging (DWI)

DWI data were acquired on the same 3 T PET/MRI scanner using two protocols. Scans collected prior to 2024 employed a multi-shell sequence with seven b-values (0, 25, 50, 100, 250, 500, 1,000 s/mm^2^) across 12 diffusion directions (*N =* 20). Scans acquired after 2024 used a similar sequence with nine b-values (0, 25, 50, 75, 100, 150, 250, 500, 1,000 s/mm^2^) and 12 directions. The 2D echo-planar imaging sequence parameters included TR/TE = 7000/80.3 ms, flip angle = 90°, 2 mm slice thickness, and 4 mm spacing. Additional reverse phase-encoding images (b = 0, 1,000 s/mm^2^) were acquired to correct for susceptibility-induced distortions during preprocessing.

#### 2.2.3 PET data

For Aβ PET we used 8.1 mCi injection of 18F-florbetaben ligand and acquired data between 90–110 min. PET data were reconstructed into 5-min frames using standard methods with zero echo time attenuation correction (Johns et al., 2024).

For tau PET we used 5 mCi injection of 18F-PI-2620 ligand and acquired data between 45 and 75 min post injection. These images were also reconstructed into 5-min frames with zero echo time attenuation correction as described in (Winer et al., 2025).

### 2.3 Data preprocessing

#### 2.3.1 T1w and DWI

We used an in-house developed pipeline called *ivim_fit* (Hemachandra et al., 2026) to preprocess T1w and DWI data. This pipeline adheres to the Brain Imaging Data Structure (BIDS; (Gorgolewski et al., 2016)), a standardized format for organizing and sharing neuroimaging data. The dataset accepted by the pipeline consists of T1w and DWI images in BIDS format. Initially, we skull stripped the T1w images using the deep learning-based *SynthStrip* tool available in FSL (Smith et al., 2004; Hoopes et al., 2022), followed by bias field correction using the *N4BiasFieldCorrection* tool from ANTs (Tustison et al., 2010).

For the DWI data we first denoised using the *dwinoise* function in MRtrix (Tournier et al., 2019). We then performed motion correction by registering all b0 volumes to the first b0 using rigid-body registration via NiftyReg (Modat et al., 2010). Secondly we applied the *topup* tool in FSL (Andersson et al., 2003) to correct for field inhomogeneity using the reverse phase encoded DWI data. We then performed Eddy current correction using the *eddy* tool in FSL to mitigate distortions (Andersson and Sotiropoulos, 2016). Finally, we used the greedy tool (Yushkevich, 2019) for DWI rigid registration to T1w. See Supplementary Figure 1 for a flowchart of the workflow.

#### 2.3.2 IVIM model fitting

We utilized the *IAR_LU_biexp* fitting model, based on the fundamental IVIM biexponential principle and part of the IVIM task force 2.4 (Fan et al., 2024), within our pipeline to calculate three key IVIM metrics: D (true diffusion coefficient), D* (pseudo-diffusion coefficient), and *f* (perfusion fraction). Within each participant, we averaged these IVIM measures across all the directions to obtain an average image of D, D* and *f*. Details description of the IVIM fitting can be found under the Supplementary material.

#### 2.3.3 Feature extraction

Using T1w images, we parcellated the brain into 98 regions of interest (ROIs) employing a deep learning-based algorithm *synthseg* available in *FreeSurfer* (Billot et al., 2023). These ROIs consists of 68 cortical and 30 subcortical regions, which were then used to mask the IVIM images, allowing the calculation of mean values for each ROI, resulting in a total of 294 features per participant.

In addition to this “*fit and average*” method, we also experimented on the “*average and fit*” approach, where the diffusion signal within each ROI is first averaged and then the IVIM model is fitted for averaged values. Detailed description of this comparison can be found under Supplementary material. This comparison revealed that the “*fit and average*” approach yielded reliable feature estimates in our ADRC sample, with fewer outliers and greater hemispheric consistency. Therefore, we adopted the “*fit and average*” method for all downstream analyses.

#### 2.3.4 PET data

Processing of dynamic PET data involved motion correcting the 5-min frames and summing them as described in (Landau et al., 2023). We defined 64 cortical ROIs using *FreeSurfer* v7, based on the Desikan aparc+aseg atlas (Desikan et al., 2006), applied to each participant’s structural MRI. Intensity values were extracted from the co-registered summed PET data using these *FreeSurfer* ROIs. We utilized *FreeSurfer* parcellation for PET per established processing pipeline developed for ADRC PET data protocol. IVIM data used *SynthSeg* parcellation; this difference does not affect our findings since we performed no direct region-to-region comparisons between modalities.

#### 2.3.5 Aβ burden and Aβ status

To quantify the Aβ burden, we calculated the Standard Uptake Volume Ratio (SUVR) for a global cortical ROI, within the whole cerebellum as the reference region. These SUVRs were used to covert to centiloids (Klunk et al., 2015), a continuous measure of Aβ accumulation (Rowe et al., 2017). Finally, we determined Aβ status (positive or negative) using a cutoff value of 18 centiloids (Royse et al., 2021).

#### 2.3.6 Tau burden and tau status

SUVRs for tau PET images were calculated using an inferior cerebellum reference region, defined on participants’ structural MRIs with the aid of the spatially unbiased atlas template toolbox in MATLAB (Diedrichsen et al., 2011). To represent tau burden, we created a “meta-ROI” by calculating the volume-weighted mean SUVR within specific *FreeSurfer* regions associated with pathological tau accumulation. These regions included the bilateral entorhinal, amygdala, parahippocampal, fusiform, inferior temporal, and middle temporal areas (Jack et al., 2017). This volume-weighted mean SUVR serves as the measure of tau burden for each participant. Finally, the tau status was calculated using a similar approach as described in (Young et al., 2024).

### 2.4 Data analysis

To evaluate the utilization of IVIM parameters to measure brain waste clearance, the following methods were used as described below.

#### 2.4.1 Feature selection

To select the most important IVIM features from 294 candidates (D, D*, and *f* measures from 98 ROIs), we used binary Aβ status (positive/negative) as the target variable because it represents a clinically relevant classification in neurodegenerative disease. Critically, the model was trained only on this binary classification without access to information about regional protein distribution, burden magnitude, or spatial patterns of accumulation. This approach allowed us to test whether IVIM metrics could independently identify brain regions that are clinically meaningful for protein pathology, providing an unbiased assessment of which IVIM features are most informative for detecting Aβ-related pathophysiology.

**Figure 2:**
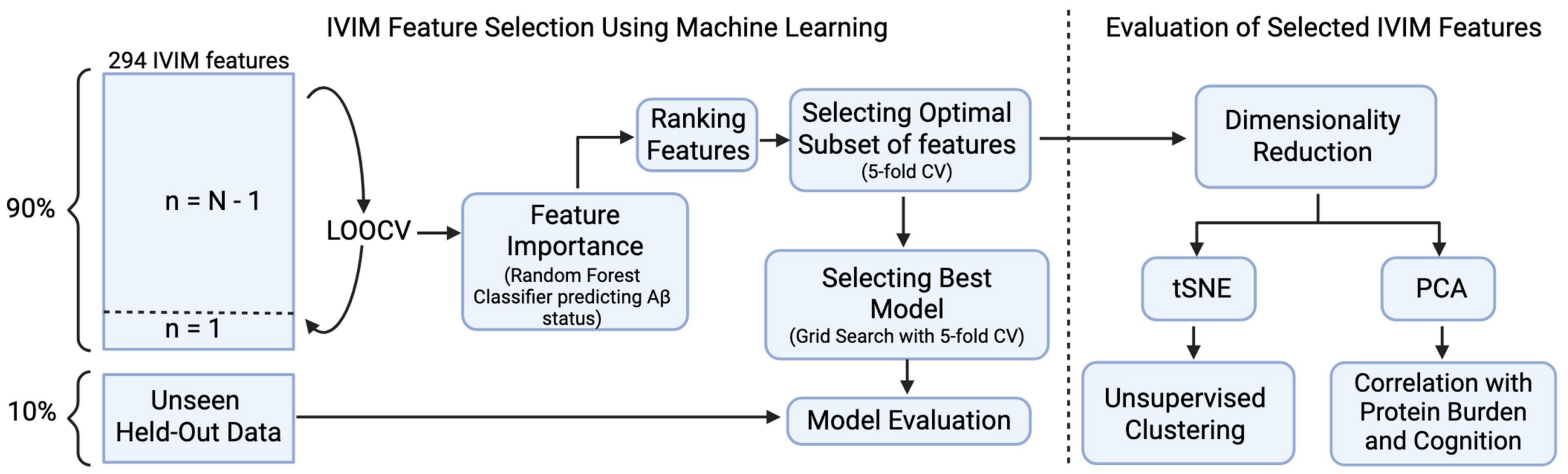
Schematic diagram of the feature selection and evaluation process. The dataset was split into 90% training and 10% held-out sets. A random forest classifier was employed to be trained to predict amyloid status and to estimate the feature importance. Feature importance estimation was performed within Leave-One-Out Cross-Validation. Features were then ranked by their mean importance and an optimal feature subset was selected via accuracy-based evaluation. This subset features were used in retraining to find a best model to evaluate the held-out dataset. Selected features were also used in dimensionality reduction using both tSNE and PCA for unsupervised clustering and to study correlation with protein burden.

First, all IVIM data were split into a training set (90%) and a held-out validation set (10%) using stratified random sampling to maintain the proportional balance of Aβ-positive and Aβ-negative participants in both sets. A Random Forest classifier (Breiman, 2001) estimated feature importance within a Leave-One-Out Cross-Validation (LOOCV) framework, which was chosen to maximize training data utilization given the modest sample size. Feature importance was calculated as the mean decrease in classification accuracy when each feature was randomly permuted, with scores averaged across LOOCV folds. The optimal number of features was determined by ranking features by their importance scores and systematically evaluating 5-fold cross-validation accuracy across feature subsets of varying sizes, a wrapper-based feature selection approach (Elisseeff, 2003) that balances predictive performance with model parsimony and interpretability. Accuracy was calculated as the proportion of correctly classified cases (true positives and true negatives) divided by the total number of cases. Conceptually, accuracy reflects a weighted average of sensitivity and specificity, where the weighting depends on the proportion of positive and negative cases in the dataset. Given the aim of this study was to identify the most informative features rather than to develop a diagnostic model, accuracy provided a simple and interpretable measure of model performance. The Random Forest model was retrained on the optimal feature subset using the training dataset (with 5-fold CV) and evaluated on the held-out validation dataset. Features were standardized using training data parameters. The held-out 10% served solely as an independent validation set to verify that the selected features generalize to unseen data, it was never used for any training, feature selection, or hyperparameter tuning decisions. Figure 2 is a schematic diagram that summarizes the feature selection workflow, and a detailed description of the process can be found under the Supplementary material.

#### 2.4.2 Unsupervised clustering and alignment with clinical categories

The set of selected features were used in dimensionality reduction using both principal component analysis (PCA) and t-distributed stochastic neighbor embedding (t-SNE) to evaluate whether IVIM-derived features could naturally separate participants based on their clinical etiology without explicit supervised labeling. This unsupervised clustering approach was employed to assess the intrinsic discriminative power of IVIM metrics and to visualize potential subgroup structure within the data. We leveraged the complementary strengths of both methods: PCA, a linear dimensionality reduction technique, provides an interpretable, variance-maximizing projection suitable for downstream statistical analyses and reveals global data structure. In contrast, t-SNE—a nonlinear method—was applied exclusively for cluster visualization, as it excels at preserving local structures and revealing subtle, nonlinear relationships that are often obscured in PCA. Visual inspection of the resulting clusters was performed to determine whether participants with similar clinical category get grouped in the reduced-dimensional space, thereby validating the biological relevance of the selected IVIM features. While PCA ensures reproducibility and global data representation, t-SNE facilitates intuitive exploration of cluster topology (Maaten and Hinton, 2008; Jolliffe and Cadima, 2016).

#### 2.4.3 Comparative analysis of IVIM with PET and its association with cognition

Finally, we evaluated associations between IVIM metrics, PET derived continuous measures of protein accumulation, and cognition as a validation step. Dimensionality reduced IVIM features, i.e., PCA components 1 to 3 were used in this analysis. First, each PCA component was compared with Aβ burden and tau burden by fitting linear models. PCA components showing the most significant correlations were then selected to compare with MoCA scores. Sensitivity analyses were also performed using partial correlations controlling for age and sex to assess the robustness of observed relationships. By examining whether features selected based solely on binary amyloid classification also correlate with continuous amyloid and tau burden, and cognitive performance, we sought to establish whether these IVIM parameters capture biologically meaningful information about protein pathology rather than non-specific or artifactual signals.

## 3 Results

The average age of our 94 participants was 72.4 ± 9.5, 45 of whom were Aβ positive. Regarding the clinical consensus diagnoses, our cohort included 32 neurologically normal individuals, 27 with LBD, 28 with AD, and 7 with other neurological diagnoses. Of those, 46 participants were cognitively unimpaired, 37 with mild cognitive impairment, 8 with Dementia and 3 with other cognitive etiologies (traumatic brain injury, alcoholism, bipolar and depression). See Table 1 for complete demographic data.

**Table 1:** Demographic and clinical characteristics by Aβ status.

Variable	All	Aβ negative	Aβ positive	*P*-value
*N*	94	49	45	–
Age (years), mean (SD)	72.43 (9.47)	70.99 (9.28)	73.99 (9.52)	0.126
Sex, *n* (%)				
M	56 (59.6%)	33 (67.3%)	23 (51.1%)	0.164
F	38 (40.4%)	16 (32.7%)	22 (48.9%)	
MoCA score, mean (SD)	23.57 (4.77)	25.21 (3.10)	21.77 (5.59)	<0.001
Race, *n* (%)				
Asian	10 (10.6%)	5 (10.2%)	5 (11.1%)	0.566
Black or African American	2 (2.1%)	1 (2.0%)	1 (2.2%)	
More Than One Race	1 (1.1%)	0 (0.0%)	1 (2.2%)	
Unknown/Not Reported	2 (2.1%)	2 (4.1%)	0 (0.0%)	
White	79 (84.0%)	41 (83.7%)	38 (84.4%)	
Ethnicity, *n* (%)				
Hispanic or Latino	9 (9.6%)	8 (16.3%)	1 (2.2%)	0.039
NOT Hispanic or Latino	84 (89.4%)	40 (81.6%)	44 (97.8%)	
Unknown / Not Reported	1 (1.1%)	1 (2.0%)	0 (0.0%)	
Cognitive group, *n* (%)				
CU	46 (48.9%)	33 (67.3%)	13 (28.9%)	<0.001
MCI	37 (39.4%)	14 (28.6%)	23 (51.1%)	
Dementia	8 (8.5%)	0 (0.0%)	8 (17.8%)	
Other	3 (3.2%)	2 (4.1%)^a,b^	1 (2.2%)^c^	
Protein burden				
A β , mean (SD)	39.21 (47.75)	3.94 (7.78)	77.62 (43.11)	<0.001
A β burden, range	−19.00–188.00	−19.00–17.00	18.00–188.00	
Tau burden, mean (SD)	1.39 (0.48)	1.19 (0.09)	1.61 (0.62)	<0.001
Tau burden, range	0.94–3.83	0.94–1.47	0.97–3.83	
Tau status, *n* (%)				
Positive	24 (25.5%)	–	–	
Negative	70 (74.5%)	–	–	

Continuous variables are presented as mean (SD). Categorical variables are presented as *n* (%). *P*-values are from independent *t*-tests for continuous variables and chi-square tests for categorical variables. CU, Cognitively Unimpaired; MCI, Mild Cognitive Impairment; Other: Traumatic Brain Injury and Alcoholism^a^, Depression^b^ and Bipolar^c^.

### 3.1 Feature selection

The Random Forest classifier ranked all 294 IVIM features by their importance scores measured during predicting amyloid status with LOOCV. Feature subsets of varying sizes were evaluated, and the top 25 features yielding the highest cross-validation accuracy (79.7%). When evaluated on the held-out dataset, this 25-feature model achieved 80.0% predictive accuracy for amyloid status.

**Figure 3:**
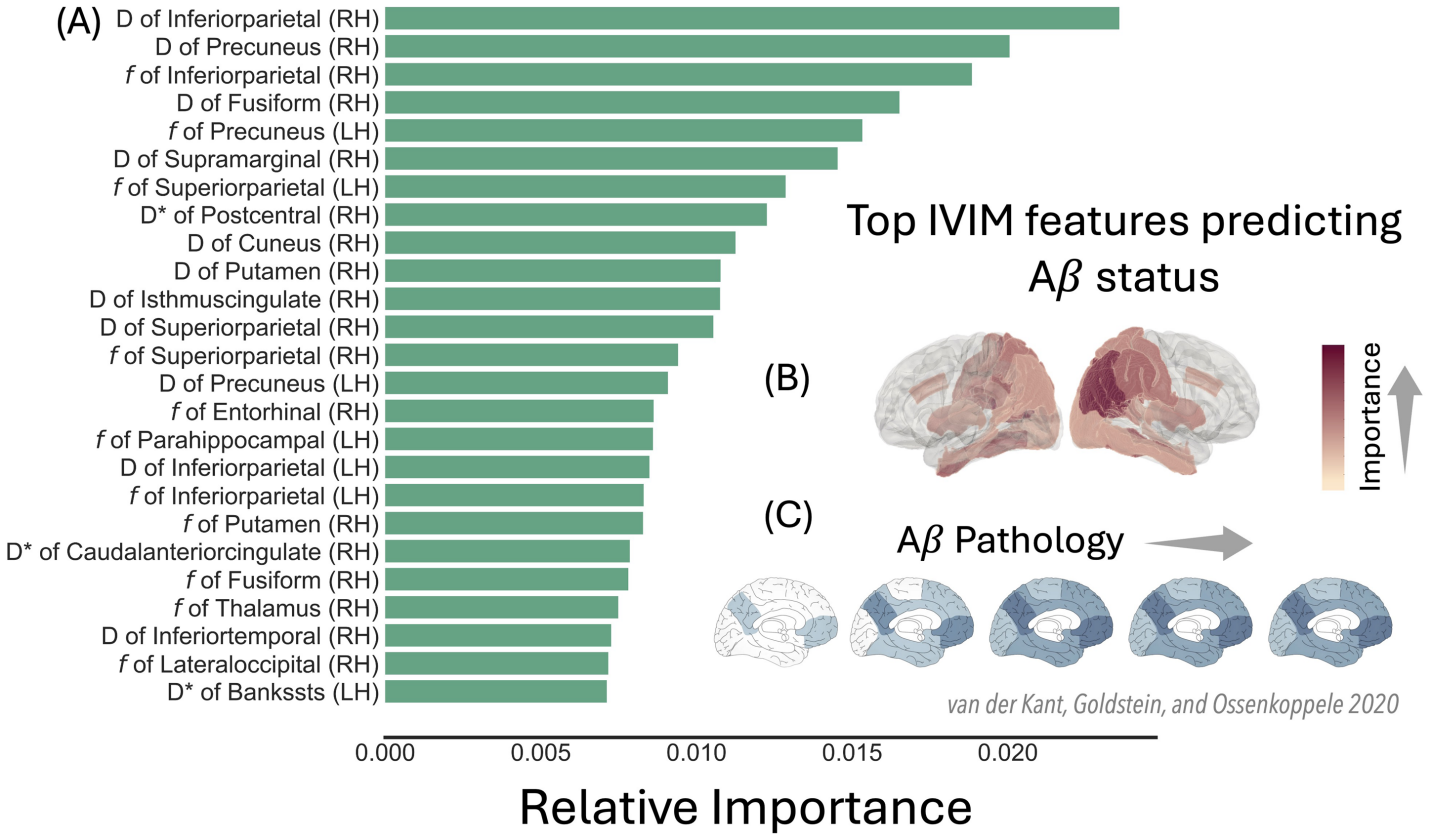
Important IVIM features that predict Aβ status and the corresponding brain regions. **(A)** Bar plot displaying the top 25 important features; feature names are formatted as “feature of region” (RH: right hemisphere, LH: left hemisphere). **(B)** Brain image highlighting the important regions. **(C)** Previously reported brain regions displaying the spread of Aβ pathology for comparison. IVIM metrics: D (true diffusion), D* (pseudo-diffusion coefficient), and *f* (perfusion fraction). Source: https://tinyurl.com/4p3dc3sy

Figure 3A shows the selected IVIM features ranked by their relative significance and includes all three IVIM metrics, with D and f being the most prominent features. Figure 3B presents a brain image highlighting the 25 brain regions where these key features are identified.

### 3.2 Unsupervised clustering and alignment with clinical categories

**Figure 4:**
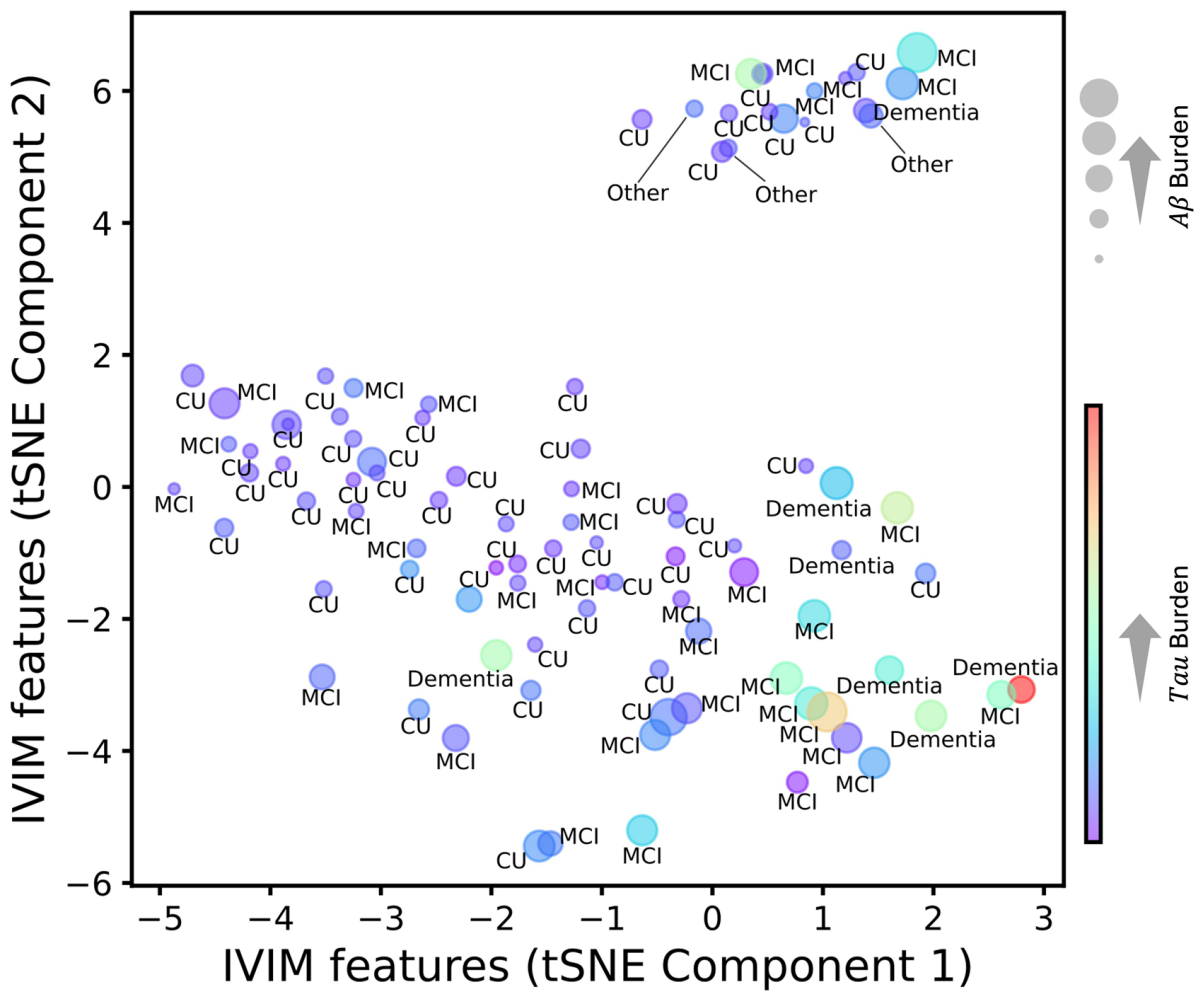
Unsupervised clustering of the data using the dimensionality reduced IVIM features using t-distributed stochastic neighbor embedding (t-SNE). Dot size indicates Aβ burden, and color represents Tau burden. Unlabeled dots represent cognitively unimpaired participants. CU, Cognitively Unimpaired; MCI, Mild Cognitive Impairment, Other: traumatic brain injury, alcoholism, bipolar and depression.

The 25 selected features were utilized for unsupervised clustering, reducing the data dimensions to two t-SNE components (Figure 4) and showing two primary clusters: one smaller, compact cluster and one larger cluster. Based on this observation, we applied K-means clustering with (k = 2) to the original high-dimensional data, resulting in two well-defined clusters. These clusters aligned with the visual groupings observed in the t-SNE visualization, with a Silhouette Score of 0.4, indicating reasonable grouping by the K-Means algorithm (Rousseeuw, 1987). Overlaying clinical diagnosis on these clusters showed distinct patterns, with cognitively normal individuals predominantly (83%) in the larger cluster and MCI/Dementia participants more dispersed (see section “Discussion”).

### 3.3 Comparative analysis of IVIM with PET (Aβ and tau) and its association with cognition

**Figure 5:**
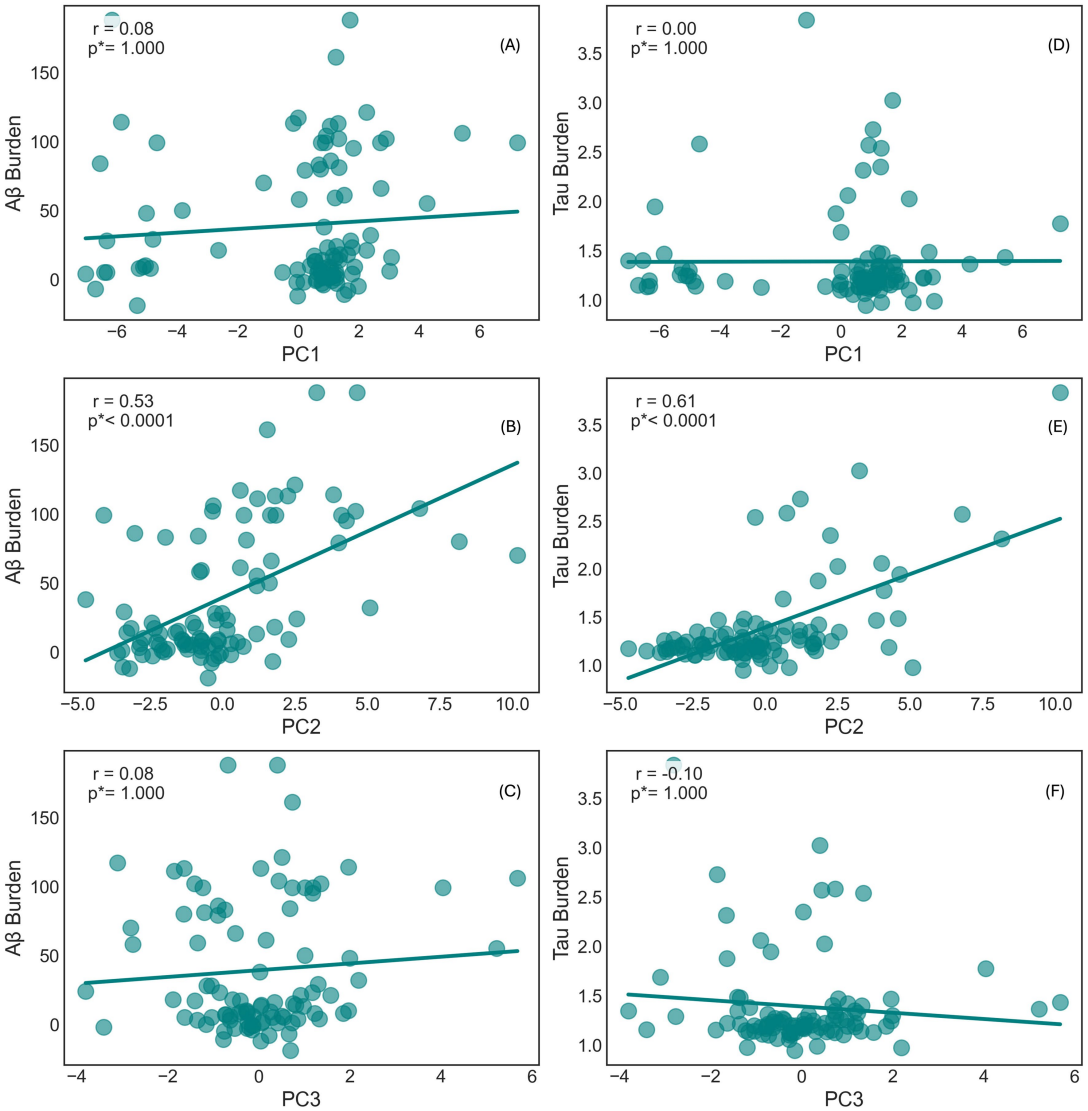
Relationships between IVIM-derived PCA components and protein burden measures. Scatter plots showing correlations between principal component analysis (PCA) components derived from IVIM features and amyloid-beta (Aβ) and tau protein burden. **(A)** PC1 vs. Aβ burden, **(B)** PC2 vs. Aβ burden, **(C)** PC3 vs. Aβ burden, **(D)** PC1 vs. tau burden, **(E)** PC2 vs. tau burden, **(F)** PC3 vs. tau burden. Each panel displays individual data points with a linear regression fit line (solid line). Pearson’s correlation coefficient (r) and Bonferroni-corrected *p*-values (P*) are shown in each panel to indicate the strength and statistical significance of the relationships.

The 25 selected features were used in a PCA analysis. We focused on the top 3 principal components, which cumulatively explained 71% of the variance (PC1: 34%, PC2: 28%, PC3: 9%), capturing the majority of information while maintaining interpretability. See Supplementary material for more details on component selection and PCA loadings (Supplementary Figure 5). PC1 was dominated by f from parietal association cortices (inferior parietal, superior parietal, precuneus), reflecting regional variations in microvascular perfusion. PC2 was dominated by D from posterior cortical and temporal regions, including the precuneus, inferior and superior parietal cortex, fusiform gyrus, and multiple temporal areas. PC3 showed mixed contributions from perfusion-related parameters (D*, f) across scattered subcortical and cortical regions without a clear spatial pattern. We correlated each of these components with Aβ burden and tau burden (Figure 5). Only PC2 showed a significant correlation with Aβ burden (r = 0.53, p* < 0.0001) and tau burden (r = 0.61, p* < 0.0001) and they remained significant when controlling for age and sex (partial r = 0.52 and 0.64, respectively, both p* < 0.0001). See Supplementary Table 1 for more details.

**Figure 6:**
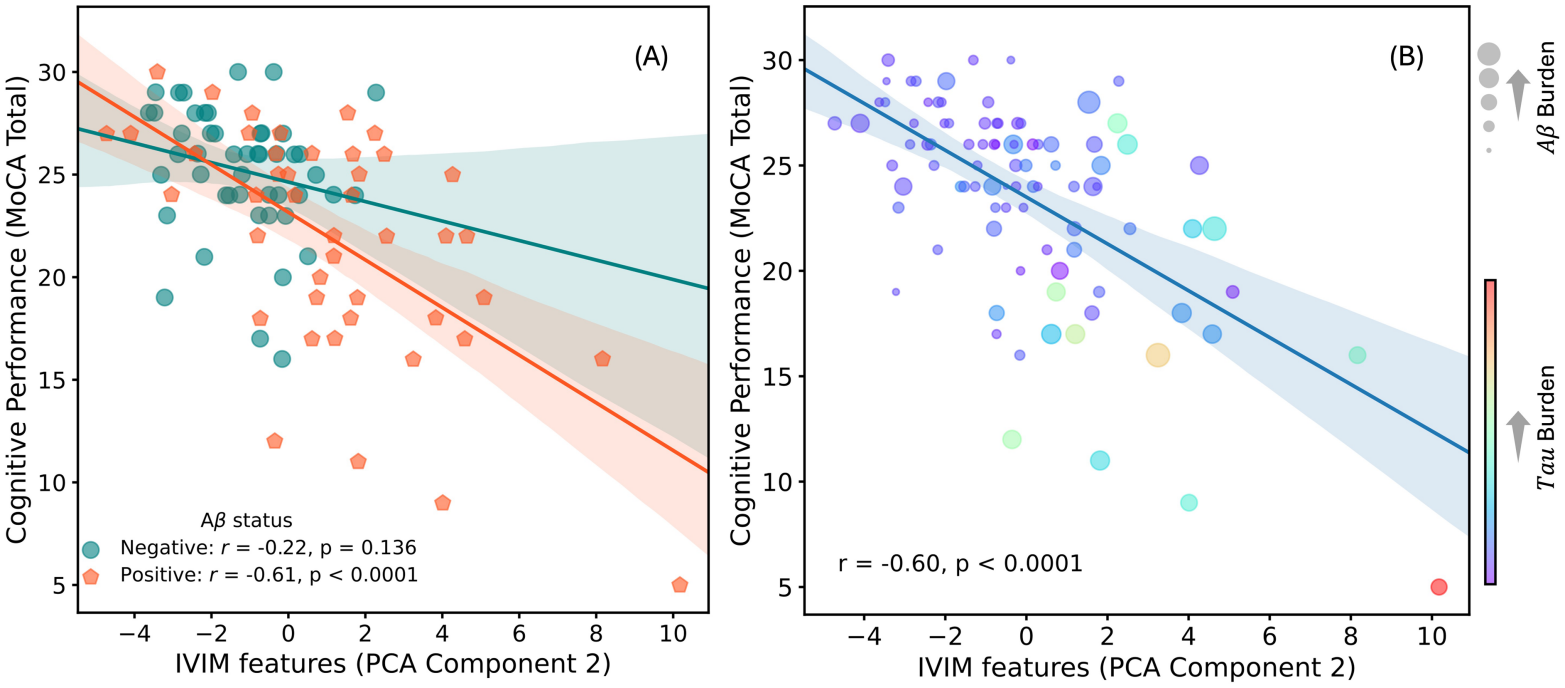
Correlation plot of PCA component 2 (from selected IVIM features) versus Cognitive Performance (MoCA scores). **(A)** Displays MoCA total vs. PCA component 2, stratified by the Aβ status and **(B)**, same data, where amyloid and tau burden overlayed (dot size indicates Aβ burden, and color represents Tau burden). Lower MoCA scores indicate greater cognitive decline.

To understand the clinical meaning to these relationships, we correlated PC2 with MoCA, stratified by Aβ status. First, we found a significant negative correlation between MoCA and PC2 using the combined Aβ groups (r = −0.60, p* < 0.0001) (Figure 6B). This remained significant when controlled for age and sex (r = −0.59, p* < 0.0001). When stratified by Aβ status the correlation between MoCA and PC2 was only significant in the Aβ positive group (r = −0.61, p* < 0.0001), but not in the Aβ negative group (r = −0.22, *p* = 0.136; Figure 6A). In Figure 6B the overlayed Aβ and tau burdens visually illustrate that the protein burden is following the observed trend of worse cognition (lower MoCA) with higher burden.

## 4 Discussion

In this study, we investigated IVIM features as proxy measures for brain waste clearance dysfunction by determining the relationship between noninvasive diffusion MRI metrics derived from IVIM modeling and the accumulation of pathological proteins in the brain measured by PET imaging. Random Forest feature selection identified 25 key IVIM features that predicted Aβ status with 80.0% accuracy, with PC2 of these features showing significant correlations with both Aβ burden (r = 0.53, *p <* 0.0001) and tau burden (r = 0.61, *p <* 0.0001). Importantly, PC2 correlated negatively with MoCA scores specifically in Aβ-positive individuals (r = −0.61, *p <* 0.0001). Hence, providing methodological insights for IVIM processing as well as providing evidence for a link between IVIM metrics and pathological protein accumulations that are associated with neurodegenerative diseases of aging.

### 4.1 Association of IVIM with protein accumulation, clinical category and cognition

We hypothesized IVIM metrics would relate to pathological protein accumulation in neurodegenerative aging. Using Random Forest classification as a feature selection tool, we identified 25 IVIM features that predicted amyloid status with 80.0% accuracy on held-out data. While the primary purpose was feature selection rather than diagnostic development, this accuracy validates that IVIM captures biologically meaningful signals related to amyloid pathology. The anatomical distribution of important IVIM features localized predominantly in parietal regions, aligned with brain areas traditionally affected by Aβ in Alzheimer’s disease (van der Kant et al., 2020), as illustrated in Figure 3C. This spatial correspondence was further supported by significant correlations between IVIM metrics and both Aβ (r = 0.53, *p <* 0.0001) and tau burden (r = 0.61, *p <* 0.0001). To our knowledge, this is the first study to directly correlate PET-measured protein pathology with IVIM parameters in humans using simultaneous PET/MRI. While prior work has theoretically linked IVIM to glymphatic function based on fluid dynamics principles, our findings provide the first empirical evidence of associations between IVIM metrics and protein accumulation in neurodegeneration. Although consistent with the hypothesis that altered fluid dynamics contribute to impaired protein clearance, we acknowledge that correlation alone cannot establish causal mechanisms.

In addition to regional amyloid co-localization, we studied whether IVIM metrics were related to clinical groups. Specifically, we used an unsupervised clustering analysis to find distinct clinical groups based on their IVIM features. The larger cluster in Figure 4 shows enrichment for cognitively normal individuals (approximately 83% of all CN participants cluster here), forming a noticeable grouping. Participants with MCI and dementia tend to be positioned at the periphery. Interestingly, people with a high protein burden, who were diagnosed with dementia or mild cognitive impairment grouped relatively closely together in this cluster. This pattern could suggest that as protein pathology increases, IVIM signatures diverge progressively from the “normal” pattern. There was not a specific disease pattern in the smaller cluster; interestingly, all 3 participants with “other” cognitive etiologies such as traumatic brain injury, alcoholism, bipolar and depression clustered in this group. Further investigation is needed to understand the physiological differences between these two groups of individuals that we detected with IVIM diffusivity characteristics.

Our PCA analysis provided insight into which patterns of IVIM variance relate to protein pathology. Despite PC1 explaining the largest proportion of variance (33.67%) in the amyloid-selected IVIM features, it showed no significant associations with amyloid or tau burden. PC1 was dominated by perfusion fraction (*f*) in parietal association cortices, distinguishing amyloid-positive from amyloid-negative groups on average. This could explain why these features were selected by the machine learning classifier but do not scale linearly with protein burden magnitude. This suggests that perfusion alterations captured by PC1 likely reflect binary state differences rather than graded changes that parallel disease severity. In contrast, PC2, characterized dominantly by true diffusion coefficient (D), demonstrated robust correlations with both amyloid and tau burden. This observed associations could reflect specific biological relationships between tissue microstructure in vulnerable brain regions (precuneus, parietal, and temporal cortex) and protein accumulation that aligns well with established patterns of amyloid accumulation in Alzheimer’s disease, providing biological plausibility for the observed associations.

Given the significant correlation between PC2 and both Aβ and tau burden, we investigated whether this component also related to cognitive impairment, a key clinical manifestation of neurodegeneration. PC2 showed a significant negative correlation with MoCA scores, indicating that IVIM patterns associated with greater protein burden also corresponded to worse cognitive performance. Notably, this relationship was specific to Aβ-positive individuals and absent in Aβ-negative participants, suggesting that the link between altered fluid dynamics and cognition is driven by protein pathology rather than normal aging. In amyloid-positive individuals, protein accumulation appears to be linked to tissue microstructural changes (captured by IVIM) and cognitive decline through shared neurodegenerative processes. In amyloid-negative individuals, tissue microstructural variations were not related to cognitive performance and likely reflect normal aging heterogeneity or other processes that do not engage the specific cascade linking protein pathology to cognitive impairment. Together, these findings reveal a convergent relationship between IVIM-measured fluid dynamics, protein accumulation, and cognitive decline, warranting further investigation into how this relationship contribute to functional decline in neurodegeneration.

### 4.2 Limitations

Despite the promising findings of this analysis, several limitations must be acknowledged. First, the study’s reliance on a relatively small sample of 94 participants may limit the generalizability of the results, particularly in diverse populations. Additionally, the cross-sectional design restricts the ability to infer causality between IVIM metrics and protein accumulation or cognitive impairment. While our amyloid positive and negative groups were well matched on age and sex, there could be other confounding factors, such as variations in participant demographics and comorbidities, was not specifically considered and could have influence the observed relationships. Our feature selection was based solely on amyloid status due to the limited sample size of tau-positive participants. This raises the question of whether different IVIM features would be selected if tau status were used as the target variable. Future studies with larger samples across diverse tauopathies and disease stages will be needed to determine whether distinct IVIM signatures emerge for different proteinopathies. Furthermore, the methodological differences between the *Fit and Average* and *Average and Fit* approaches may introduce variability in the data. Although pseudo-diffusion estimates appear to be sensitive to the fitting method and D* maps are typically noisier than D and *f* maps (Wu et al., 2015; Paschoal et al., 2018), D* may still provide valuable information for glymphatic imaging. For example, Rane Levendovszky et al. (2024) found reduced D* with sleep deprivation that may have implication for ISF solute transport, and in our study, regional D* was among the IVIM features selected in relation to protein burden. However, it is important to note that IVIM does not directly measure glymphatic function, as it cannot isolate cerebrospinal fluid (CSF) or interstitial fluid (ISF) dynamics alone but rather captures a composite signal reflecting CSF, ISF, and blood flow. Therefore, while IVIM metrics may serve as indirect proxies for brain fluid dynamics relevant to waste clearance, they should be interpreted cautiously as surrogate markers rather than direct measures of glymphatic activity. Lastly, while the study employs advanced imaging techniques, the inherent limitations of diffusion MRI and PET imaging, such as low resolution and sensitivity, may affect the accuracy of the measurements and interpretations.

## 5 Conclusion

In conclusion, our study highlights methodological considerations in IVIM analyses and implicates IVIM as a measure to study protein accumulation in neurodegenerative diseases. The integration of advanced imaging techniques, such as simultaneous PET-MR scans, represents a unique opportunity to elucidate some of the mechanisms that link pathological protein accumulation and cognitive impairment, paving the way for future research aimed at developing targeted therapeutic strategies.

## Data Availability

The raw data supporting the conclusions of this article will be made available by the authors pending final legal and ethical review, without undue reservation.
