# Supplementary material for: Brain protein burden is related to intravoxel incoherent motion: PET-MR imaging study
Source: Front Neurosci. 2026 Jun 16;20:1841093. doi: 10.3389/fnins.2026.1841093 (PMC13314920; doi:10.3389/fnins.2026.1841093)
Supplement: Supplementary file 1 [file Data_Sheet_1.docx]

Supplementary Material

**1. IVIM Parameter Estimation**

To estimate the intravoxel incoherent motion (IVIM) parameters, we employed the *IAR_LU_biexp* fitting model, part of the IVIM task force 2.4, utilizing python libraries. The model fits the signal decay data to the bi-exponential IVIM equation:

$$S\left( b \right)=S_{0}[fexp\left( -bD* \right)+\left( 1-f \right)\exp\left( -bD \right)]$$

where S(b) is the signal intensity at b-value b, S0 is the signal intensity without diffusion weighting, *f* is the perfusion fraction, D∗ is the pseudo-diffusion coefficient associated with perfusion, and D is the diffusion coefficient.

The fitting process was performed using a nonlinear least-squares (NLLS) approach via the *curve_fit* function from the SciPy library. The data were normalized by their maximum value to stabilize the fitting process, with the fitted S_0_ parameter rescaled post-fitting to reflect the original signal magnitude. The model parameters (S_0_, f, D∗, D) were constrained within user-defined bounds to ensure physically plausible estimates. Default bounds were set as follows: S_0_ ∈ [0, ∞), f ∈ [0, 1], D∗ ∈ [0.005, 0.1] mm^2^/s, and D ∈ [0, 0.004] mm^2^/s. Initial parameter guesses were set to S_0_ = 1, f = 0.2, D∗ = 0.03 mm^2^/s, and D = 0.001 mm^2^/s.

To accommodate different units, an optional rescaling feature was included to convert the diffusion coefficients (D∗ and D) from mm^2^/s to µm^2^/ms, adjusting both the initial guesses and bounds accordingly. This ensured compatibility with b-values provided in µm^2^/ms. The fitting procedure was applied on a voxel-by-voxel basis using the *multi_voxel_fit* decorator in DIPY library (Garyfallidis et al. 2014), enabling efficient processing of multi-voxel datasets. The maximum number of function evaluations was set to 10,000 to ensure convergence.

**2. Image Preprocessing Workflow (IVIM_fit)**

T1w and DWI data were processed using an in-house developed pipeline, as described by (Hemachandra et al. 2026) This pipeline adheres to the Brain Imaging Data Structure (BIDS), a standardized format for organizing and sharing neuroimaging data. The dataset accepted by the pipeline consists of T1w and DWI images in BIDS format. Initially, T1w images underwent skull stripping using the deep learning-based *SynthStrip* tool available in FSL (Smith et al. 2004; Hoopes et al. 2022) Following this, bias field correction was performed using the *N4BiasFieldCorrection* tool from ANTs (Tustison et al. 2010).

For DWI data, the initial step involved denoising using the *dwidenoise* function in MRtrix (Tournier et al. 2019). A motion correction was performed by registering all b0 volumes to the first b0 using rigid-body registration via NiftyReg (Modat et al. 2010). Subsequently, the *topup* tool in FSL was applied to correct for field inhomogeneity, utilizing the reverse phase encoding data that was available (Andersson, Skare, and Ashburner 2003). Finally, Eddy current correction was performed using the *eddy* tool in FSL to mitigate distortions caused by eddy currents (Andersson & Skare, 2002). Figure 1s is a schematic diagram depicting the main preprocessing steps within the *IVIM_fit* pipeline.


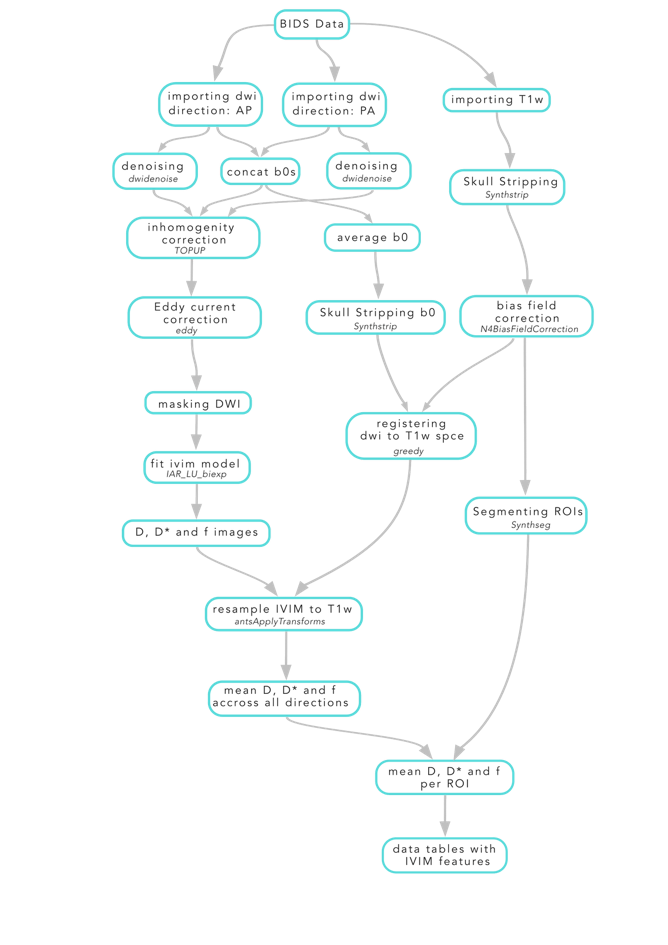


Figure 1s. Schematic diagram of the workflow of the *IVIM_fit* pipeline.

**3. Feature Selection Using Machine Learning**

We developed a machine learning pipeline to classify amyloid status (positive or negative, based on centiloid values) using Intravoxel Incoherent Motion (IVIM) neuroimaging data. The pipeline aimed to identify the optimal subset of predictive features and evaluate the impact of perfusion-related features on classification accuracy. The dataset was split into 90% training and 10% held-out sets. A Random Forest (RF) classifier was employed for its robustness and feature importance estimation. The process involved three stages: (1) feature importance estimation using Leave-One-Out Cross-Validation (LOOCV), (2) optimal feature subset selection via accuracy-based evaluation, and (3) retraining and evaluation on the held-out dataset.

**Data Preparation**

The IVIM dataset comprised samples with neuroimaging features and binary amyloid status labels. The data were randomly split into a 90% training set and a 10% held-out set for final evaluation, ensuring no data leakage. All features were standardized using z-scoring, with standardization parameters (mean and standard deviation) computed on the training data only.

**Stage 1: Feature Importance Estimation**

For each feature set, feature importance was estimated using an RF classifier within an LOOCV framework. An initial RF model was used with default parameters in *sklearn* python library.

In each LOOCV fold, the training subset (N-1 samples) was standardized, and the test sample was transformed using the training subset’s parameters. The RF model was trained on the standardized training subset, and feature importance scores (mean decrease in impurity) were recorded. Importance scores were averaged across LOOCV folds to obtain robust estimates for each feature. True labels and positive class probability scores were collected during LOOCV for receiver operating characteristic (ROC) analysis.

**Stage 2: Optimal Feature Subset Selection**

To determine the optimal number of features, features were ranked by their average importance scores. Subsets of the top 1 to k features were evaluated, where k was the minimum of 50 or the total number of features in the feature set. For each subset, an RF model was trained on the standardized training data using the best hyperparameters selected from the grid search. Parameters used for gridsearch: number of trees (50, 100, 200), maximum features per split (square root or log2), maximum depth (none, 10, 20, 30), minimum samples per split (2, 5, 10), and minimum samples per leaf (1, 2, 4). Mean accuracy was computed via 5-fold cross-validation and the subset size yielding the highest mean accuracy was selected as the optimal number of features. This data-driven approach replaced arbitrary cutoffs. Accuracy versus number of features was plotted, with the optimal number highlighted, to visualize the selection process.

**Stage 3: Model Retraining and Held-Out Evaluation**

For each feature set, an RF model was retrained on the full training dataset using the optimal feature subset. The training data was standardized, and a new grid search (with the same hyperparameter grid) was performed with 5-fold cross-validation to optimize the model for the reduced feature set. The best-performing model was selected based on mean CV accuracy.

The retrained model was evaluated on the 10% held-out dataset, standardized using the training data’s parameters. The held-out dataset included the optimal features. Performance was assessed using F1-score.

**4. Fit and Average vs Average and Fit**

We obtained ROI-based IVIM features using two methods: *Fit and Average*, where the diffusion signal is fitted to IVIM models before averaging within ROIs, and *Average and Fit*," where the signal is averaged first and then fitted to the model. We compared group differences to identify significant feature variations between these approaches. Additionally, we assessed outliers (beyond 1.5 times the interquartile range) as an indicator of data noise and performed a chi-square test for comparison. Correlation coefficients from Left (LH) and Right (RH) hemisphere IVIM assessments were analyzed using Pearson r to determine which method yields more consistent measurements across hemispheres. We also computed Pearson r values for D, D*, and *f* in relation to amyloid and tau burden to find the method more closely associated with protein accumulation. All P values for correlations were adjusted for multiple comparisons using the Bonferroni correction method. Results were summarized as the percentage of ROIs that are significant from each method (Table 1).

Table 1. Summary of statistics from the comparison between Fit and Average vs Average and Fit methods.

| Experiment | Method | *Fit and Average* | *Average and Fit* |
| --- | --- | --- | --- |
| Group difference | D | 97% | |
|  | D* | 98% | |
|  | *f* | 58% | |
| Outlier analysis | D | 1% | 3% |
|  | D* | 6% | 33% |
|  | *f* | 3% | 18% |
| LH and RH correlation | D | 91% | 87% |
|  | D* | 81% | 43% |
|  | *f* | 89% | 94% |

Group difference: Percentage of the ROIs displaying a statistically significant difference between the two methods. Outlier analysis: Percentage of the ROIs displaying significantly higher amount of outliers compared to the other method. LH and RH correlation: Percentage of ROIs displaying statistically significant correlations between LH and RH values.

Group Differences: Differences between the two methods were assessed using Mann-Whitney U tests. Among the three IVIM metrics, D showed significant differences in 97% of its features, followed by D* with 98%, and f with only 58% [Supplementary Figure 4s].

Outlier Analysis: We evaluated the prevalence of outliers for each fitting method. The Average and Fit approach yielded more outliers than the Fit and Average method. Specifically, 3% of D features were identified as outliers, followed by 33% for D* and 18% for f. In contrast, the Fit and Average method reported only 1% for D, 6% for D*, and 3% for f. These findings indicate that D* is particularly sensitive to the methodological approach, with the Average and Fit method associated with a higher incidence of outliers.

Hemispheric Correlation: In comparing left hemisphere and right hemisphere IVIM measures, the Fit and Average method demonstrated superior consistency in significant correlations. D features showed a correlation rate of 89% with Fit and Average, compared to 94% with Average and Fit. For D*, the Fit and Average method achieved an 81% correlation rate versus 43% for Average and Fit. For f, the Fit and Average method achieved 91% correlations compared to 87% for Average and Fit [Supplementary Figure 5s].

**Discussion (Fit and Average vs Average and Fit):**

The comparison between the *Fit and Average* and *Average and Fit* approaches revealed substantial differences in the extracted features, particularly for the D* metric, which exhibited the most pronounced variations and least hemispheric consistency. This suggests that the choice of analytical technique can influence the results. The higher incidence of outliers associated with the Average and Fit method further emphasizes the potential pitfalls of this approach, indicating a greater susceptibility to noise and variability in the data. Moreover, applying the *Fit and Average* analysis yielded more consistent correlations between the left and right hemispheres for D and *f* metrics, reinforcing the notion that methodological rigor is essential for reliable interpretations of imaging data. Several studies compared the IVIM data derived from these two methods. Englund et al., 2024 and Lebret et al., 2024, have reported less test-retest reliability with the data obtained from the spine using *Average and Fit* method compared to *Fit and Average*. Moreover, Lemke et al., 2011 reported significantly decreased D* values (similar to our findings) with the *Average and Fit* method and showed improved image quality with less standard deviation when applying the *Fit and Average* method to their IVIM data within liver, kidney and spleen. Overall, our results support the use of the *Fit and Average* method for this study. This conclusion applies specifically to the data and analysis parameters of this study and does not generalize regarding IVIM fitting methodologies.


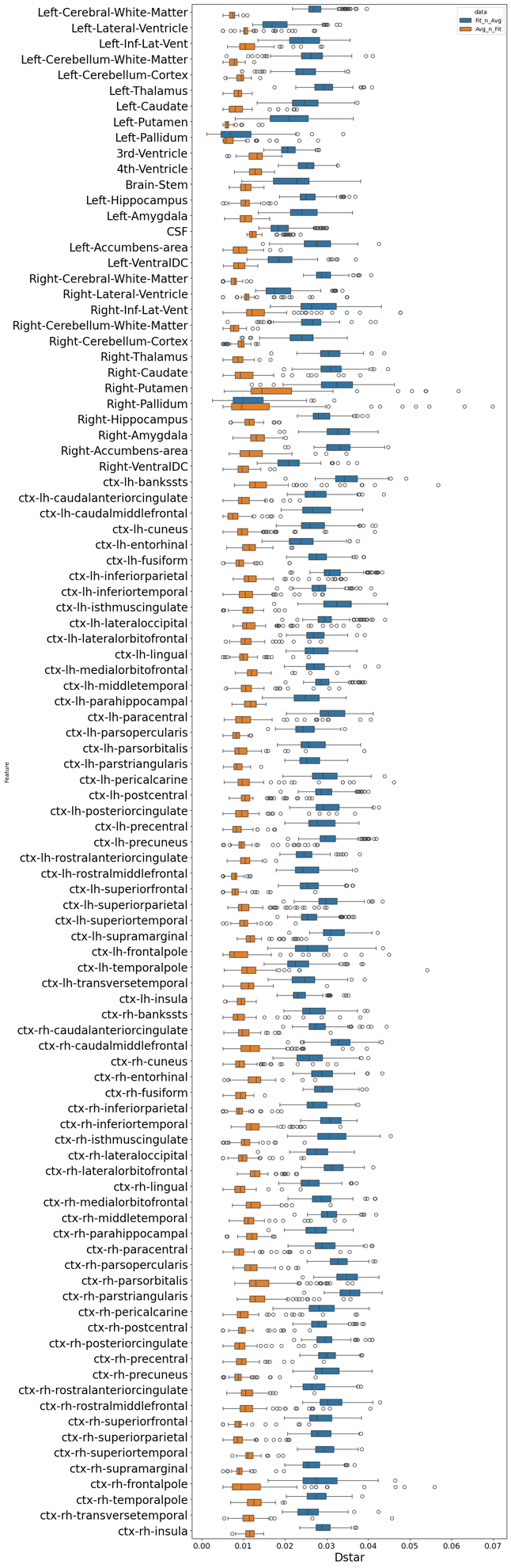


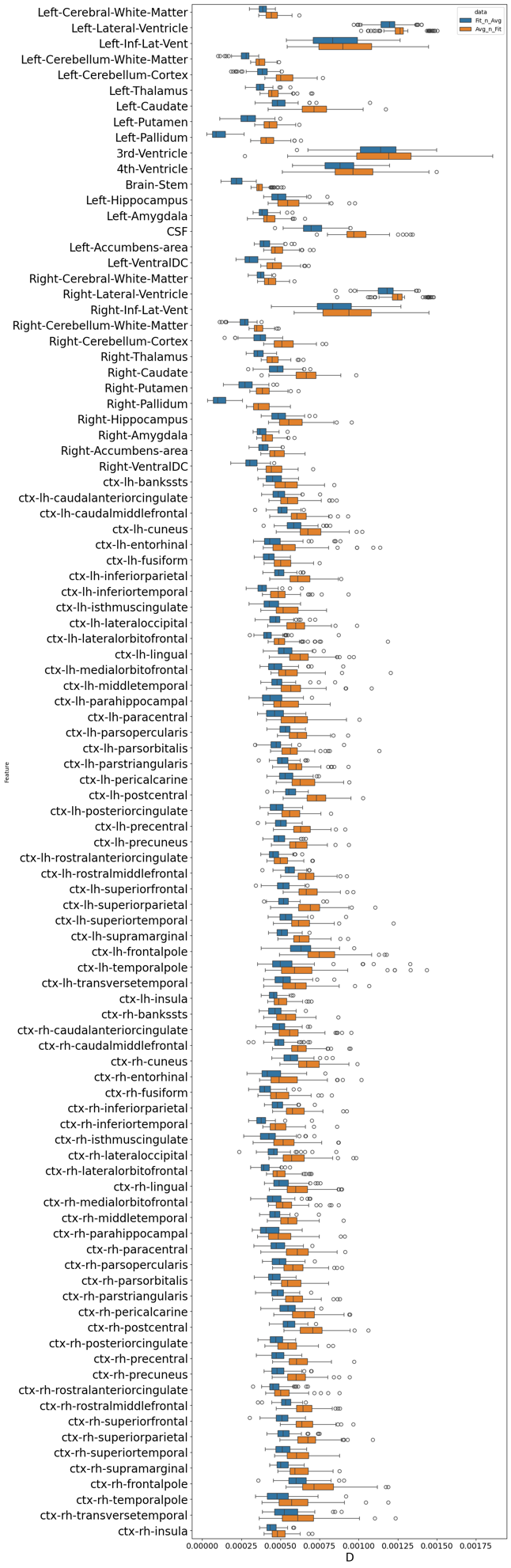

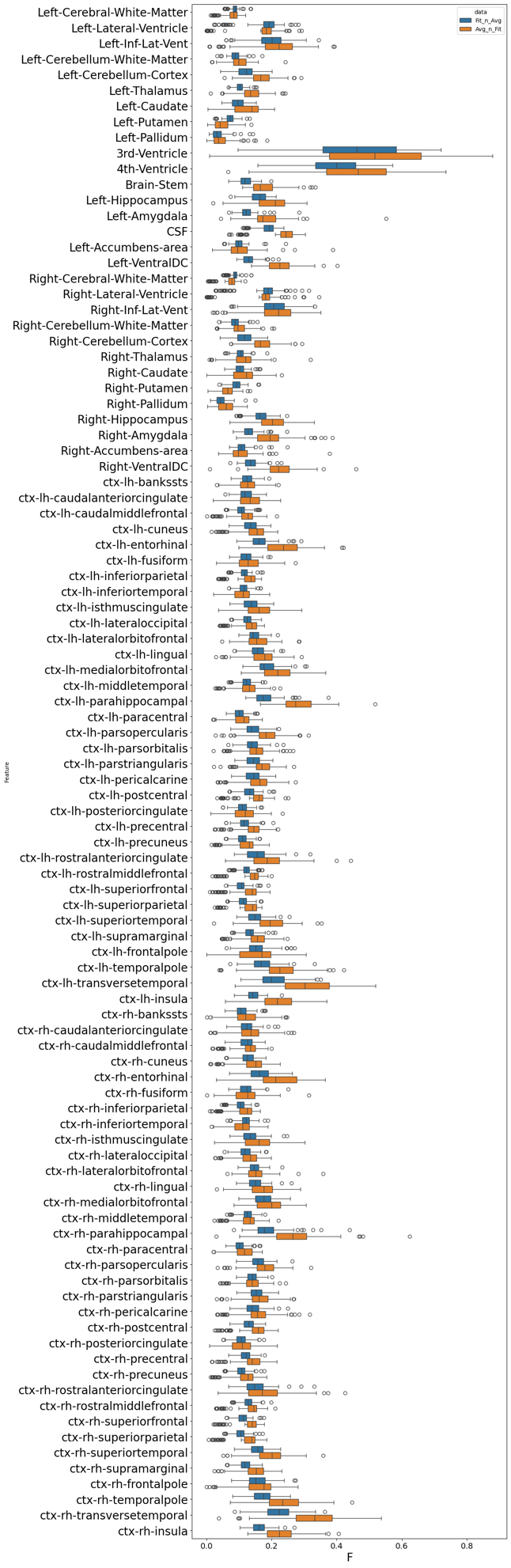


D

D*

*f*

Figure 2s. Box plots displaying the group differences between *Fit and Average* and *Average and Fit* values from the three IVIM metrics, D, D* and *f* calculated within 98. D* measures are displaying the most noticeable difference between the two methods.


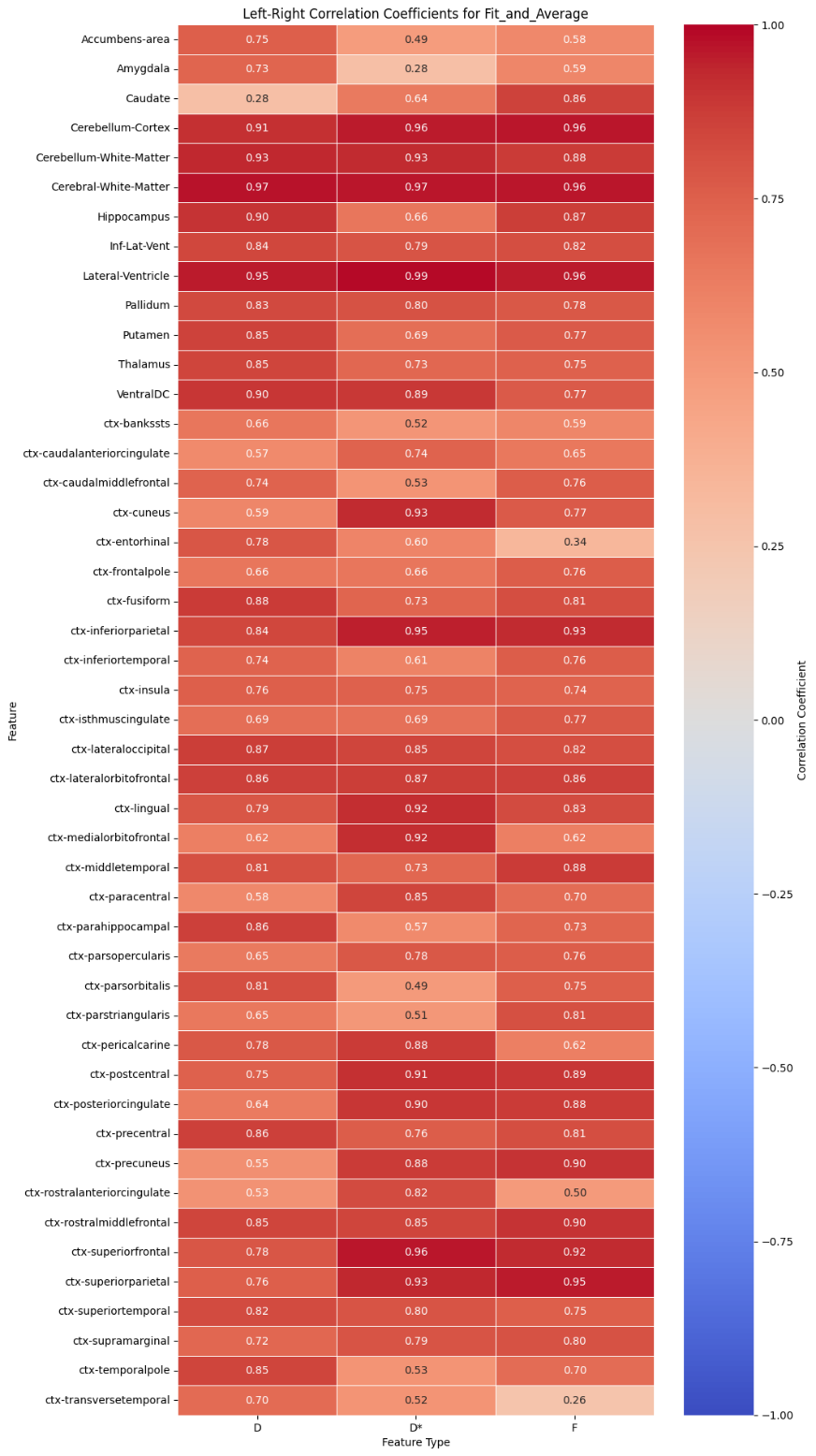

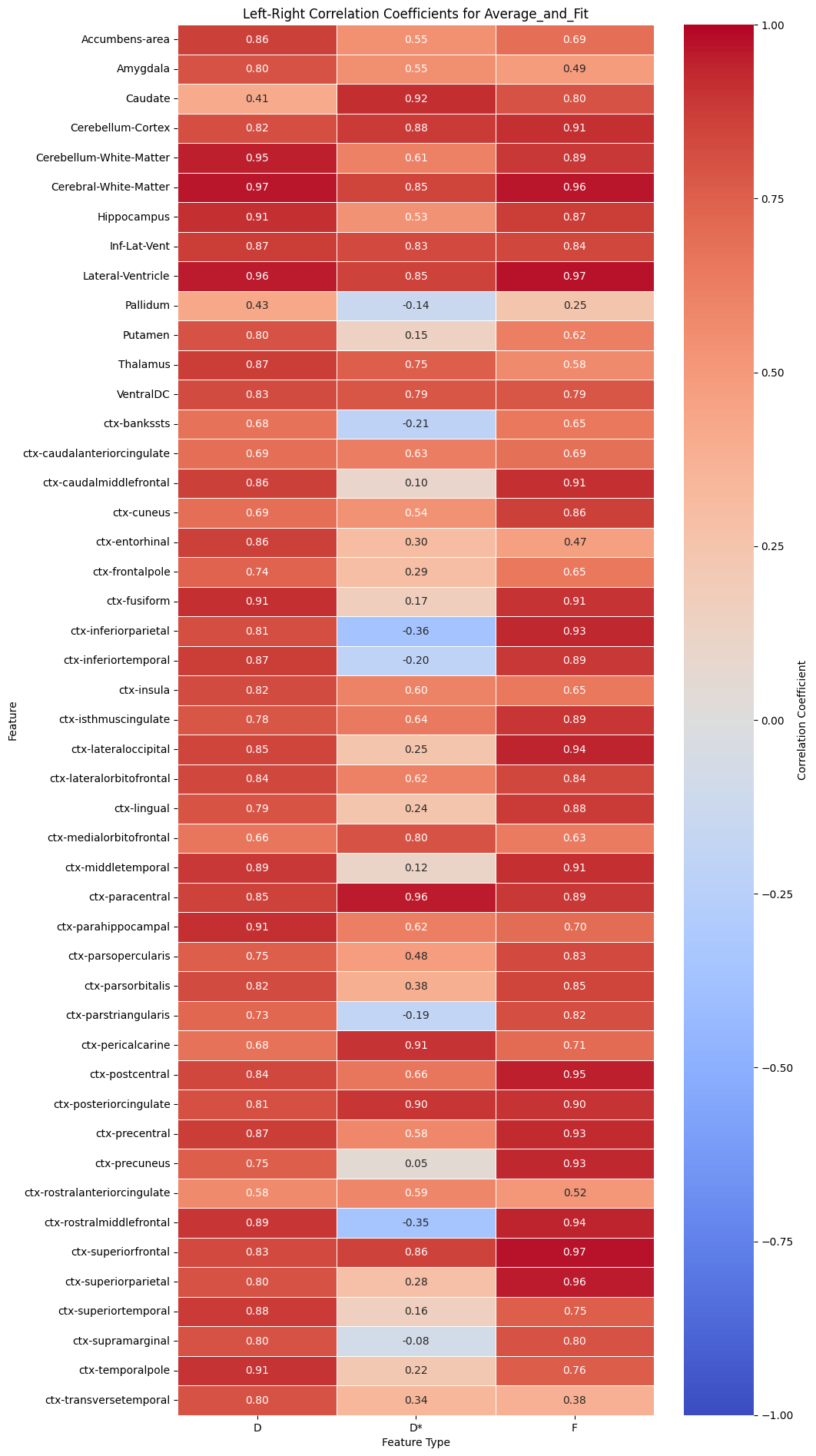


D

D*

*f*

D

D*

*f*

Figure 3s. Heatmap displaying the Pearson r correlation coefficient for the hemispheric correlation calculated for the three IVIM measures, D, D* and *f*. Left panel is displaying the values from *Average and Fit* method and the right panel is from *Fit and Average* method. Hemispheric correlation of the D* values derived from *Average and Fit* method displaying the lowest correlation values indicating the inconsistency in the values derived using that method. Values from *Fit and Average* method is displaying consistently higher hemispheric correlation across the three measures.

**5. Principal Component Analysis**

**Methodology and Component Selection**

Principal Component Analysis (PCA) was applied to the 25 IVIM features selected through Random Forest classification to reduce dimensionality while retaining the majority of variance. We selected the top 3 principal components based on cumulative variance explained and scree plot analysis. The top 3 components cumulatively explained 71% of the total variance (PC1: 33.67%, PC2: 27.53%, PC3: 8.93%), and visual inspection of the scree plot (Figure 4s) revealed an elbow after the third component, with subsequent components each explaining less than 5% of variance. This approach balances variance retention with model parsimony, consistent with standard PCA practice in neuroimaging research.

**Biological Characterization of Principal Components**

To understand what each principal component represents biologically, we examined the feature loadings (Figure 5s). PC1 was dominated by perfusion fraction (*f*) from parietal association cortices including the inferior parietal cortex, superior parietal cortex, precuneus, and thalamus, reflecting regional variations in microvascular perfusion. PC2 was characterized mainly by the true diffusion coefficient (D) from posterior cortical and temporal regions including the precuneus, inferior and superior parietal cortex, fusiform gyrus, and temporal areas. These regions comprise the default mode network and are known to be vulnerable to early amyloid deposition in Alzheimer's disease. The diffusion coefficient D reflects tissue microstructural properties including neuronal density, extracellular space geometry, and membrane integrity. PC3 showed mixed contributions from perfusion-related parameters (D*, *f*) and tissue diffusion across scattered regions without a clear spatial or parametric pattern, capturing residual regional heterogeneity.


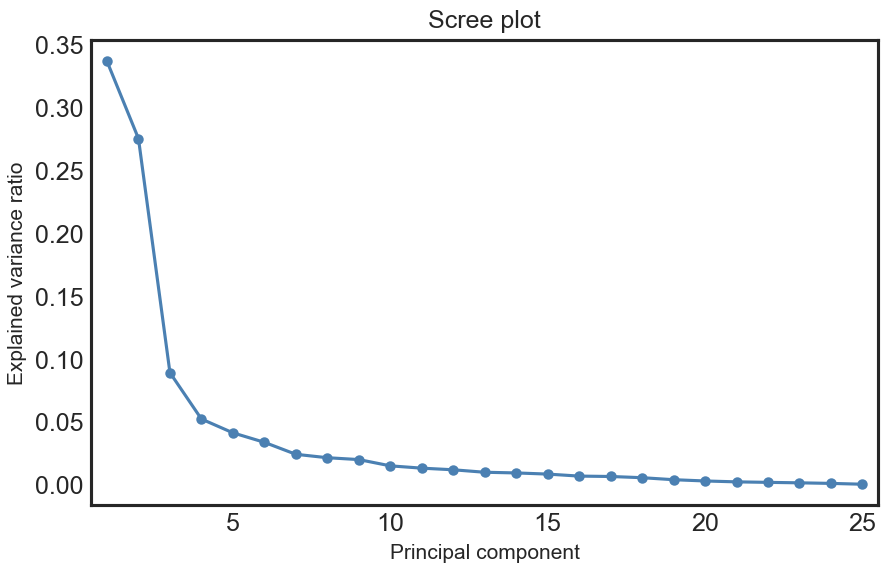


Figure 4s. Scree plot for principal component selection. Percentage of variance explained by each principal component. The top 3 components cumulatively explain 71% of variance, with an elbow visible after PC3.


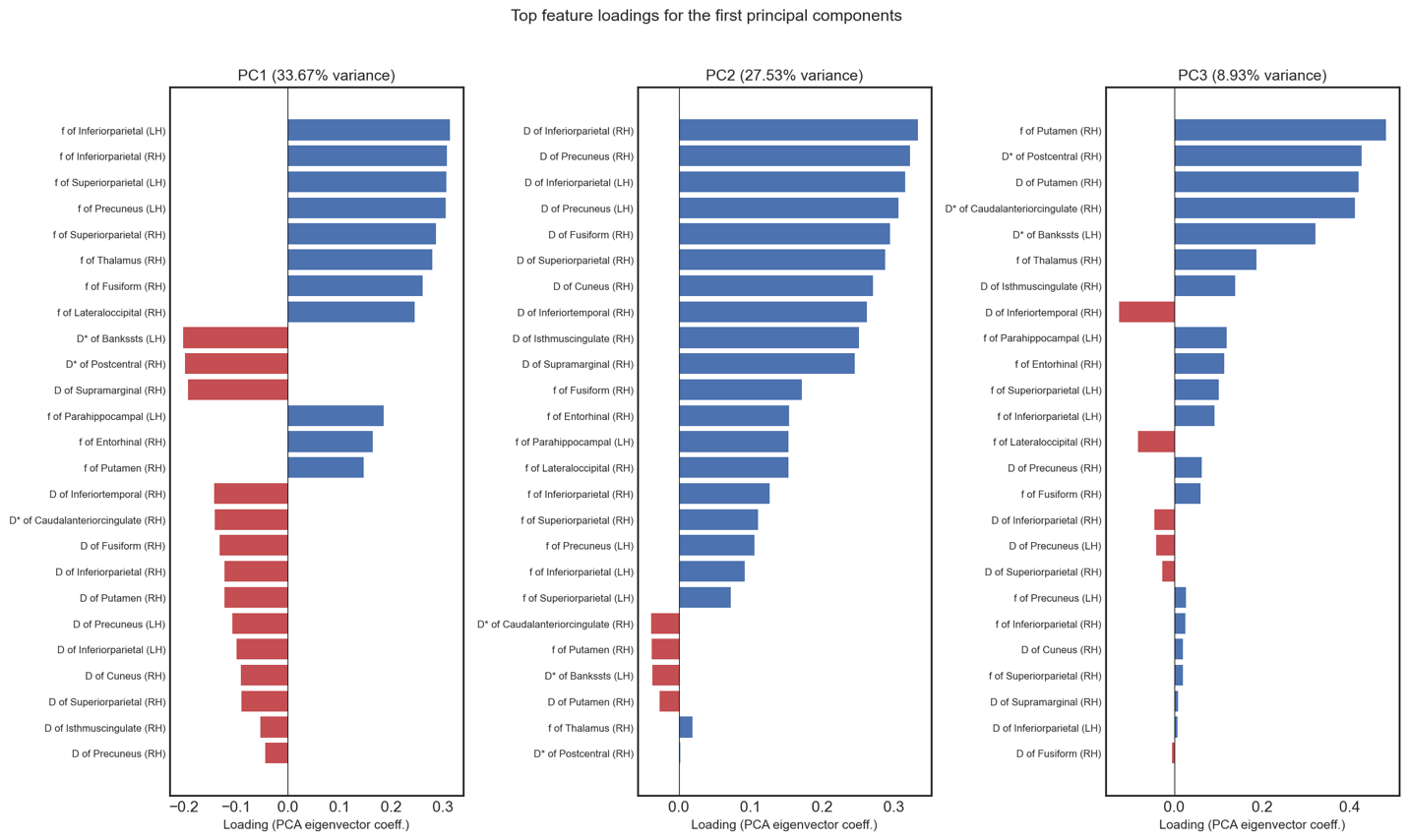


Figure 5s. Feature loadings for the top three principal components derived from the 25 selected IVIM features. Blue bars indicate positive loadings; red bars indicate negative loadings.

Independent correlation analysis of all 294 IVIM features with both amyloid (Figure 6s) and tau burden (Figure 7s) measures confirm biological specificity of PCA components. D parameters correlated significantly more strongly with protein burden than D* or *f* parameters. The spatial pattern of top correlations (precuneus, parietal, temporal regions) matches PC2 composition.


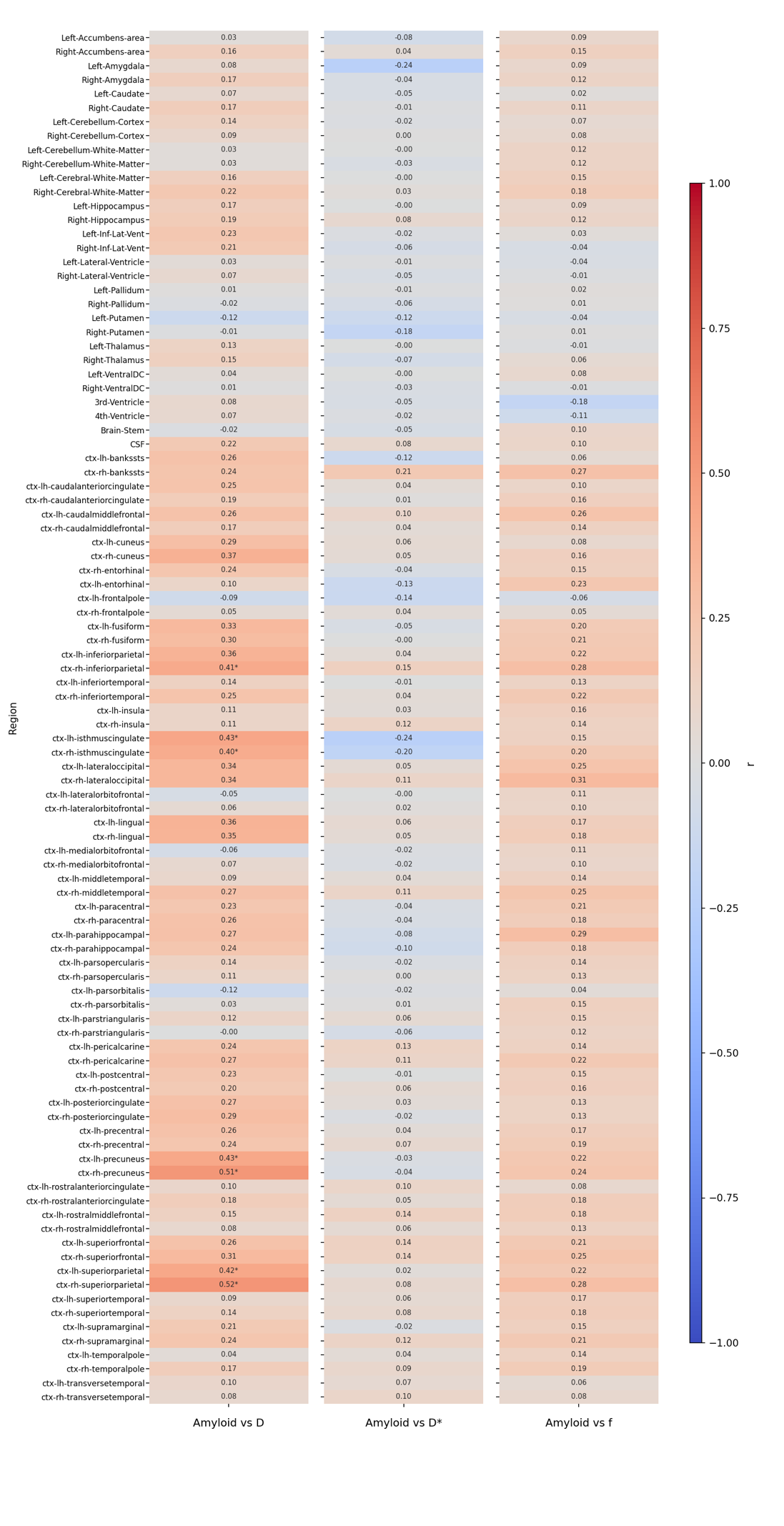


Figure 6s. Correlations between individual IVIM features and amyloid burden. Heatmap showing Pearson correlation coefficients (r) for all 294 IVIM parameters (D, D*, f across 98 brain regions) with amyloid burden. Asterisks denote significant correlations after multiple comparison correction.


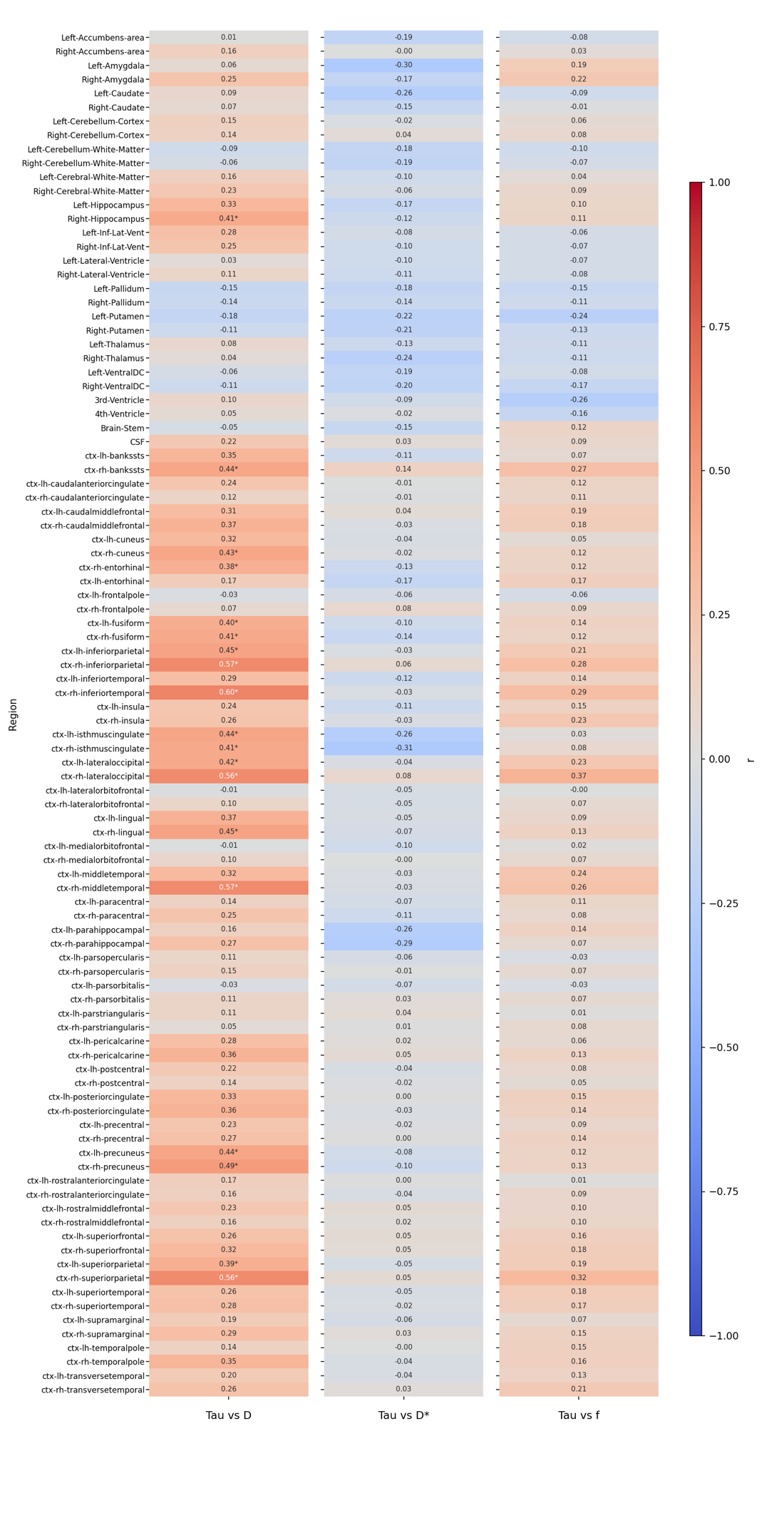


Figure 7s. Correlations between individual IVIM features and amyloid burden. Heatmap showing Pearson correlation coefficients (r) for all 294 IVIM parameters (D, D*, f across 98 brain regions) with tau burden. Asterisks denote significant correlations after multiple comparison correction.

References:

Andersson, Jesper L. R., Stefan Skare, and John Ashburner. 2003. “How to Correct Susceptibility Distortions in Spin-Echo Echo-Planar Images: Application to Diffusion Tensor Imaging.” *NeuroImage* 20 (2): 870–88.

Garyfallidis, Eleftherios, Matthew Brett, Bagrat Amirbekian, Ariel Rokem, Stefan van der Walt, Maxime Descoteaux, Ian Nimmo-Smith, and Dipy Contributors. 2014. “Dipy, a Library for the Analysis of Diffusion MRI Data.” *Frontiers in Neuroinformatics* 8 (February): 8.

Hemachandra, Dimuthu, Kevin Zheng, Eva Muller-Oehring, and Kathleen Poston. 2026. “IVIM_fit.” Zenodo. June 2026. https://doi.org/10.5281/zenodo.20560360.

Hoopes, Andrew, Jocelyn S. Mora, Adrian V. Dalca, Bruce Fischl, and Malte Hoffmann. 2022. “SynthStrip: Skull-Stripping for Any Brain Image.” *ArXiv [Eess.IV]*. arXiv. http://arxiv.org/abs/2203.09974.

Modat, Marc, Gerard R. Ridgway, Zeike A. Taylor, Manja Lehmann, Josephine Barnes, David J. Hawkes, Nick C. Fox, and Sébastien Ourselin. 2010. “Fast Free-Form Deformation Using Graphics Processing Units.” *Computer Methods and Programs in Biomedicine* 98 (3): 278–84.

Smith, Stephen M., Mark Jenkinson, Mark W. Woolrich, Christian F. Beckmann, Timothy E. J. Behrens, Heidi Johansen-Berg, Peter R. Bannister, et al. 2004. “Advances in Functional and Structural MR Image Analysis and Implementation as FSL.” *NeuroImage* 23 Suppl 1: S208-19.

Tournier, J-Donald, Robert Smith, David Raffelt, Rami Tabbara, Thijs Dhollander, Maximilian Pietsch, Daan Christiaens, Ben Jeurissen, Chun-Hung Yeh, and Alan Connelly. 2019. “MRtrix3: A Fast, Flexible and Open Software Framework for Medical Image Processing and Visualisation.” *BioRxiv*. bioRxiv. https://doi.org/10.1101/551739.

Tustison, Nicholas J., Brian B. Avants, Philip A. Cook, Yuanjie Zheng, Alexander Egan, Paul A. Yushkevich, and James C. Gee. 2010. “N4ITK: Improved N3 Bias Correction.” *IEEE Transactions on Medical Imaging* 29 (6): 1310–20.
