# Supplementary figures and images for: Brain protein burden is related to intravoxel incoherent motion: PET-MR imaging study
Source: Front Neurosci. 2026 Jun 16;20:1841093. doi: 10.3389/fnins.2026.1841093 (PMC13314920; doi:10.3389/fnins.2026.1841093)

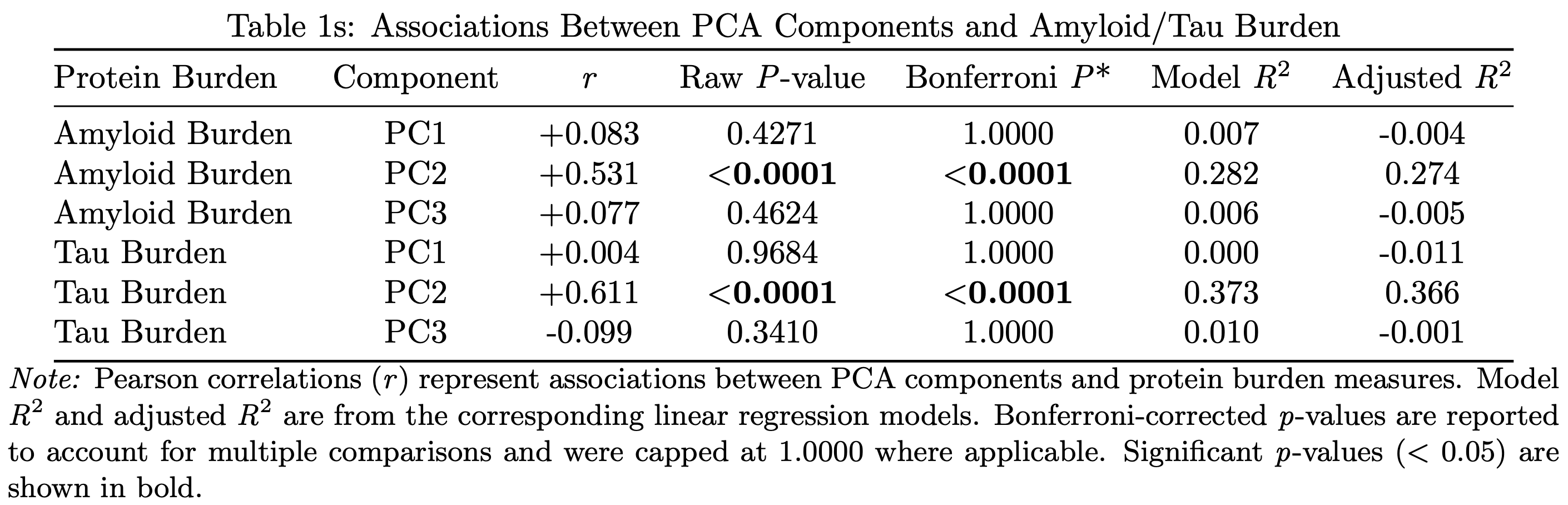

Supplement: Supplementary file 2 [file Supplementary_file_1.png]
